# Effects of donor smoking history on early post-transplant lung function measured by oscillometry

**DOI:** 10.3389/fmed.2024.1328395

**Published:** 2024-04-09

**Authors:** Natalia Belousova, Albert Cheng, John Matelski, Anastasiia Vasileva, Joyce K. Y. Wu, Rasheed Ghany, Tereza Martinu, Clodagh M. Ryan, Chung-Wai Chow

**Affiliations:** ^1^Toronto Lung Transplant Program, Ajmera Multi-Organ Transplant Program and Division of Respirology, University Health Network, Tonronto, ON, Canada; ^2^Department of Medicine, Temerty Faculty of Medicine, University of Toronto, Toronto, ON, Canada; ^3^Pneumology, Aduch Cystic Fibrosis and Lung Transplantation Department, Foch Hospital, Suresnes, France; ^4^Biostatistics Research Unit, University Health Network, Toronto, ON, Canada; ^5^Toronto General Pulmonary Function Laboratory, University Health Network, Toronto, ON, Canada

**Keywords:** lung transplant, oscillometry, donor smoking history, donor selection, lung function

## Abstract

**Introduction:**

Prior studies assessing outcomes of lung transplants from cigarette-smoking donors found mixed results. Oscillometry, a non-invasive test of respiratory impedance, detects changes in lung function of smokers prior to diagnosis of COPD, and identifies spirometrically silent episodes of rejection post-transplant. We hypothesise that oscillometry could identify abnormalities in recipients of smoking donor lungs and discriminate from non-smoking donors.

**Methods:**

This prospective single-center cohort study analysed 233 double-lung recipients. Oscillometry was performed alongside routine conventional pulmonary function tests (PFT) post-transplant. Multivariable regression models were constructed to compare oscillometry and conventional PFT parameters between recipients of lungs from smoking vs non-smoking donors.

**Results:**

The analysis included 109 patients who received lungs from non-smokers and 124 from smokers. Multivariable analysis identified significant differences between recipients of smoking and non-smoking lungs in the oscillometric measurements R_5-19_, X_5_, AX, R_5_z and X_5_z, but no differences in %predicted FEV_1_, FEV_1_/FVC, %predicted TLC or %predicted DLCO. An analysis of the smoking group also demonstrated associations between increasing smoke exposure, quantified in pack years, and all the oscillometry parameters, but not the conventional PFT parameters.

**Conclusion:**

An interaction was identified between donor-recipient sex match and the effect of smoking. The association between donor smoking and oscillometry outcomes was significant predominantly in the female donor/female recipient group.

## 1 Introduction

Lung transplantation is a last resort treatment for advanced lung disease, generally reserved for patients whose life expectancy without transplantation is less than 1 year, or who have severe, irreversible impairment in quality of life as a result of their disease.

In most lung transplant centers, access to transplantation is limited primarily by donor supply. The “ideal donor” is considered to be a life-long non-smoker under the age of 55 years, with a clear chest x-ray, no history of chest surgery or trauma, and no evidence of infection (1). However, most centers utilize lungs from donors who fail to meet one or more of these criteria, in order to maximize the donor pool of this limited resource. Lungs from donors with a history of cigarette smoking are frequently used, especially if the estimated total exposure is less than 20 pack-years and there is no documented history of lung disease. Some studies comparing outcomes between recipients of lungs from smoking and non-smoking donors have shown reduced overall survival and increased risk of baseline allograft dysfunction (BLAD, defined as failure to achieve a post-transplant peak FEV_1_ which is at least 80% of the predicted value) in recipients of lungs from smokers (2–6). A negative impact on early post-transplant outcomes, such as PaO2/FiO2 ratio, duration of ventilation and intensive care unit (ICU) length of stay, has also been reported (7, 8). However, a review of United Network for Organ Sharing (UNOS) data found no significant impact on mortality, freedom from chronic lung allograft dysfunction (CLAD) or all-cause mortality from utilizing donors even with a heavy (≥ 20 pack year) smoking history (9). Furthermore, it has been demonstrated that the overall risk of death is higher in patients remaining on the waitlist if lungs from smoking donors are not used (4). Therefore, the overall risk of a poor outcome needs to be balanced against the risk of death on the waitlist.

Oscillometry is a non-invasive measurement of respiratory impedance that is sensitive to physiology of small airways, a region of the lung not well characterized by conventional pulmonary function tests (cPFT). Commonly investigated oscillometry parameters include the difference between resistance at 5 Hz and 19 or 20 Hz (R_5-19_ or R_5-20_), which increases with rising small airway resistance and ventilatory inhomogeneity; reactance at 5 Hz (X_5_), a value which becomes more negative with increased lung stiffness and loss of elastic recoil; and area of reactance (AX), an integrated area of reactance between X_5_ and the resonant frequency (Fres), the frequency at which the reactance curve crosses zero. AX provides a potentially more sensitive indication of changes in the elastic properties of the lung than the single-frequency value X_5_, as it provides a measure of reactance at a range of frequencies (10). A summary of changes in oscillometry parameters associated with different lung function patterns is provided in Table 1.

**Table 1:** Typical changes to oscillometry parameters associated with different obstructive and restrictive lung function patterns.

	Obstructive pattern	Restrictive pattern
R_5_	Increased	Normal or increased
R_5–19_ (R__5–20__)	Increased	Normal
X_5_	Decreased	Decreased
AX	Increased	Increased

Oscillometry has been shown to detect abnormalities in people with respiratory symptoms but normal spirometry, and to detect early chronic obstructive lung disease (COPD) prior to changes in cPFT (11–13). R_5-20_ is higher in smokers than non-smokers, even without a diagnosis of COPD (14), and is correlated with the Global Initiative for Chronic Obstructive Lung Disease (GOLD) stage and COPD assessment scores (15). A correlation has also been found between the R_5-20_, F_res_ and the wall thickness of small airways detected on endobronchial optical coherence tomography in heavy smokers and COPD patients (16). X_5_ is more negative in smokers than non-smokers, resembling values seen in COPD patients (14).

In lung transplantation, oscillometry has been proposed as a tool for monitoring graft function to complement cPFTs, based on the rationale that many of the pathological processes involved in graft dysfunction, such as acute rejection and bronchiolitis obliterans (BO), affect the small airways (17, 18), which are poorly characterized by spirometry (19, 20). Our group has shown that oscillometry can detect episodes of acute cellular rejection which were not identified by changes in cPFT (21) and that increased intra-subject variability in X_5_, AX and R_5-19_ is associated with increased risk of chronic lung allograft dysfunction (CLAD), a key cause of long-term morbidity and mortality in lung transplantation (22). Oscillometry also provides insights into the respiratory mechanics of the different phenotypes of CLAD (23, 24).

It should also be noted that spirometry early post-transplant is likely impacted by deconditioning and recent chest surgery and may not be an accurate representation actual lung tissue function (25–27). Oscillometry, on the other hand, is effort-independent.

The current study aims to examine the differences in oscillometry parameters between recipients of lungs from smoking vs. non-smoking donors. We posit that oscillometry would identify small airway obstruction in smoking lungs even where changes are not detectable on spirometry.

## 2 Methods

The prospective single-centre observational cohort study was approved by the University Health Network (UHN) Research Ethics Board (REB# 17–5,652) and began in December 2017.

### 2.1 Study participants

Patients were recruited at their first routine post-transplant visit to the PFT lab, usually within 1 week of discharge from hospital and 3–4 weeks post-transplant. All double lung transplant recipients were eligible. Single lung recipients, patients with known anastomotic issues, and those who remained hospitalized at ≥3 months after transplant were excluded.

### 2.2 Data collection

Participating patients underwent oscillometry prior to each post-transplant visit to the PFT laboratory. Routine cPFTs except diffusing capacity for carbon monoxide (DLCO) occur weekly for the first 6 weeks, bi-weekly until 3 months post-transplant, then at 6, 9, 12, 18 and 24 months, and annually thereafter. DLCO is generally not performed during the period of frequent PFT lab visits over the first 3 months post-transplant, but is performed at each routine visit thereafter. Additional visits to the PFT laboratory may be prompted by new onset respiratory symptoms or drop in lung function on home monitoring. Oscillometry is performed using the Tremoflo device (model C-100, Thorasys, Montreal, Canada) following published technical, quality control and assurance guidelines (28, 29). Spirometry, body plethysmography and diffusing capacity are performed following international guidelines (30–32).

The primary outcomes of interest included the oscillometry parameters R_5_, R_5-19_, X_5_ and AX, as well as R_5_z and X_5_z, defined as the number of standard deviations from the normal population mean for R_5_ and X_5_, respectively. R_5-19_ rather than R_5-20_ was used, as the Tremoflo, and other devices employing the forced oscillometry technique, measure at prime number frequencies to avoid harmonic amplification. Conventional PFT parameters of interest were those known to be associated with graft function following lung transplant, specifically FEV_1_ (expressed as % predicted), ratio of FEV_1_ to forced vital capacity (FEV_1_/FVC), total lung capacity expressed as % predicted (TLC % predicted) and DLCO. CLAD-free survival was assessed as a secondary outcome.

Clinical characteristics were extracted from the Toronto Lung Transplant Program (TLTP) database, which maintains lifelong records of all patients transplanted by the programme. CLAD was defined according to the 2019 ISHLT consensus statement as a sustained (≥ 3 months) fall in FEV_1_ to below 80% of the post-transplant baseline value. Baseline is defined as the average of the two highest post-transplant FEV_1_ readings, measured at least 3 weeks apart (33).

Donor smoking history was extracted from TLTP database. This information was collected from donor families during the donation process, and recorded in the database as a binary variable, with “Yes” indicating any amount of current or prior cigarette smoking at the time of donation, and wherever possible with an estimated number of pack years. We excluded patients if donor smoking history was not recorded, or not quantified. Included recipients had a minimum of 3 months of follow up at the censor date of March 15, 2020, i.e., transplanted before December 15, 2019.

### 2.3 Statistical analysis

Baseline characteristics were compared using Fisher’s Exact Test for categorical variables and Mann–Whitney U test for continuous variables.

The oscillometry and cPFT outcomes over time were compared between smoking and non-smoking donors, and against smoking severity as quantified by pack-years, using linear mixed effects models. Covariates shown to have a statistically significant univariate association with the outcome were included in the construction of multivariable models.

Multivariable linear regression models were constructed for each outcome of interest. All models included random slopes for each participant to account for repeated measures. The candidate adjustment variables assessed for potential interaction were: recipient age, donor age, recipient body mass index (BMI), primary lung disease, *ex vivo* lung perfusion (EVLP) use, total ischemic time, sex match, ICU length of stay and transplant hospitalization length of stay. Predictors were assessed for either an additive and/or interactive effect with donor cigarette use as a binary predictor, or total donor cigarette exposure, measured in pack-years, as a continuous predictor.

The detection of additive effect indicates that the predictor has an effect on the outcome of interest, but does not influence the relationship between the outcome and the key predictor being studied, in this case, the donor smoking history. An interaction effect indicates that the variable changes the relationship between smoking history and the outcome.

To the end of obtaining accurate adjusted effect estimates for the donor smoking exposures relative to each outcome variable, great care had to be taken in identifying potential confounders and addressing possible interaction terms; there was no *a priori* reason to suppose that the same model specification would be adequate/appropriate for all outcome variables. To address this, for each combination of predictor and outcome, we fit both an additive and an interaction model. Using anova F-tests, each predictor was classified as having either an interaction effect, if the interaction model differed significantly from the null model; an additive effect only if the additive model (but not the interaction model) differed from the null model; and none if no significant effects were found.

For each outcome, a final multivariable model was fit using these findings. Heatmaps were created to visualize patterns in covariate inclusion, while varying the outcome variable (Supplementary Figure S1). Predictors were assessed for multicollinearity and those with a generalized variance inflation factor (GVIF^1/(2·df)^) > 5 were removed in a descending stepwise manner until all GVIF^1/(2·df)^ values were < 5.

Results were expressed as number-of-standard-deviations change in the outcome metric for each standard deviation increase in the predictor variable (continuous variables), with the exception of time post-transplant, where increments of 1 month were used, and donor pack years, which were analysed in 10-pack-year increments. For categorical variables, standard-deviation changes in outcome metrics were shown in comparison to the reference group.

Survival curves were constructed using the Kaplan-Myer method for comparison of CLAD-free survival between the smoking and non-smoking donor groups.

Analysis was performed using R Studio version 4.0.3.

## 3 Results

### 3.1 Enrolment and follow-up

**Figure 1:**
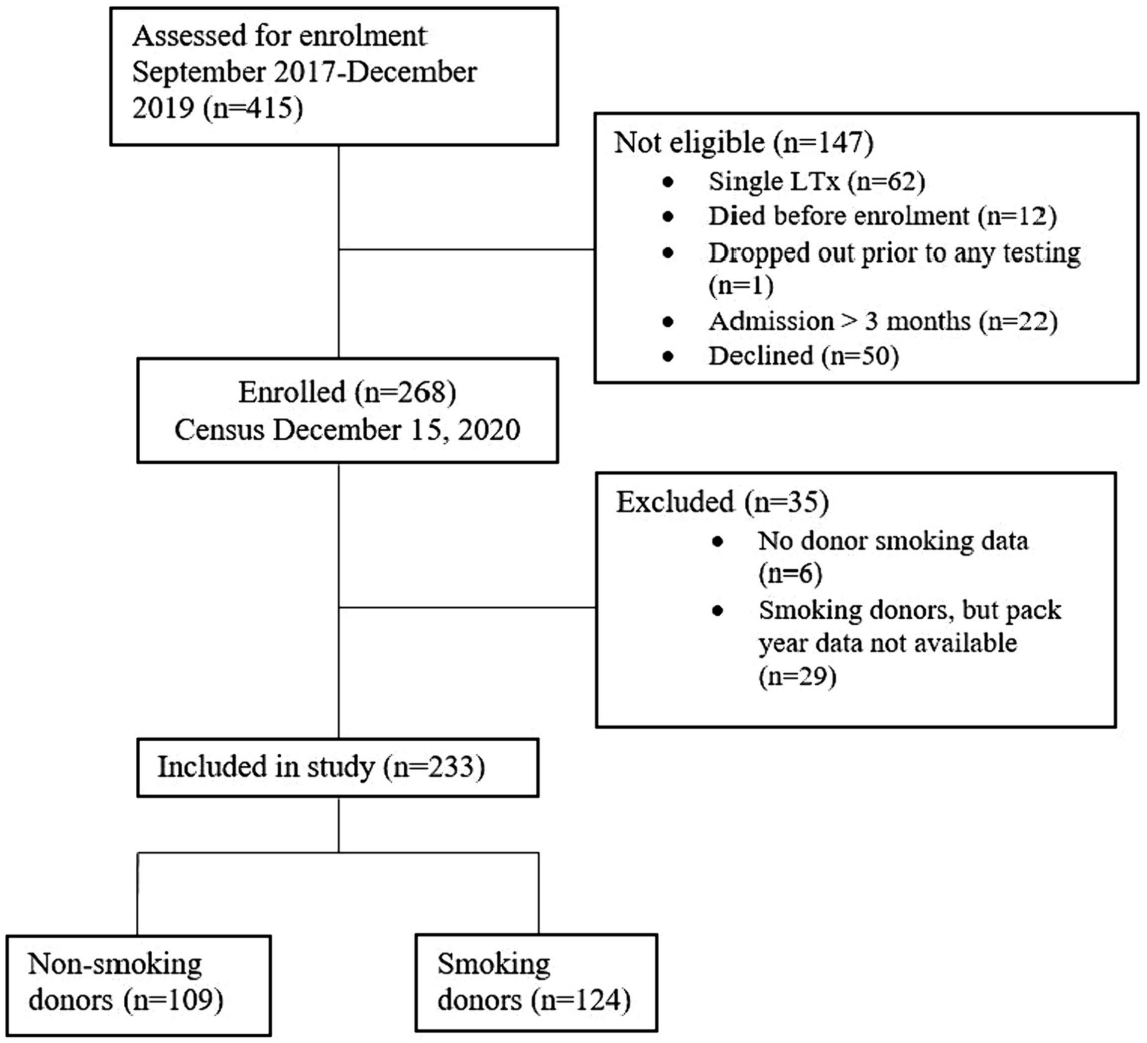
Patient selection and exclusion.

The study enrolled 268 of the 415 recipients transplanted from September 2017 to December 15, 2019. Fifty patients declined the study and 97 did not meet inclusion criteria. Thirty-five patients were excluded due to insufficient donor smoking history data. Of the 233 patients included for analysis, 109 received lungs from non-smoking donors and 124 from smoking donors (Figure 1). Median duration of follow-up was 356 days.

### 3.2 Baseline characteristics

Baseline characteristics were similar between the two groups (Table 2). Notable exceptions include more male recipients in the smoking donor group, and differences in the sex-match distribution between the groups. Specifically, in the non-smoking donor group, there was a higher proportion of F➔F sex-match (female donor and female recipient) and a lower proportion of F➔M. Among the smoking donors, the median estimated number of pack years was 10, with 38 participants receiving lungs from donors with a history of 20 or more pack years.

**Table 2:** Baseline and peri-transplant characteristics.

	Total (*n* = 233)	Non-smoking donor (*n* = 109)	Smoking donor (*n* = 124)	*p*
Recipient characteristics				
Male sex	123 (52.8)	47 (43.1)	76 (61.3)	**0.006**
Age at transplant, years	60 [46–66]	59 [47–66]	60 [46–66]	0.849
Primary disease				0.654
Cystic Fibrosis	28 (12.0)	14 (12.8)	14 (11.3)	
Emphysema/COPD	74 (31.8)	33 (30.3)	41 (33.1)	
Interstitial lung disease	95 (40.8)	42 (38.5)	53 (42.7)	
Other	36 (15.5)	20 (18.3)	16 (12.9)	
BMI at transplant, kg/m2	25.16 [21.21–27.96]	24.41 [20.71–27.23]	25.84 [21.42–28.70]	0.056
Donor characteristics				
Male sex	130 (55.8)	57 (52.3)	73 (58.9)	0.355
DCD donor	66 (28.3)	30 (27.5)	36 (29.0)	0.884
Donor age, years	48.00 [32.00–61.00]	51.00 [29.00–65.00]	44.00 [33.00–58.00]	0.150
CMV match				0.120
CMV mismatch	51 (21.9)	22 (20.2)	29 (23.4)	
CMV neg/neg	48 (20.6)	17 (15.6)	31 (25.0)	
CMV recipient positive	135 (57.5)	70 (64.2)	64 (51.6)	
Sex match donor/recipient				
F/F	75 (32.2)	45 (41.3)	30 (24.2)	**0.009**
M/F	35 (15.0)	17 (15.6)	18 (14.5)	
F/M	28 (12.0)	7 (6.4)	21 (16.9)	
M/M	95 (40.8)	40 (36.7)	55 (44.4)	
Transplant characteristics				
PRA status at transplant				
Negative	73 (31.3)	35 (32.1)	38 (30.6)	0.981
Positive current	132 (56.7)	61 (56.0)	71 (57.3)	
Positive historic	28 (12.0)	13 (11.9)	15 (12.1)	
Virtual crossmatch				0.381
Negative	183 (78.5)	83 (76.1)	100 (80.6)	
Positive historic	15 (6.4)	6 (5.5)	9 (7.3)	
Positive current	35 (15.0)	20 (18.3)	15 (12.1)	
Actual crossmatch*				0.645
Negative	57 (24.5)	26 (23.9)	31 (25.0)	
Not done	145 (62.2)	66 (60.6)	79 (63.7)	
Positive	31 (13.3)	17 (15.6)	14 (11.3)	
Perioperative PLEX	50 (21.5)	28 (25.7)	22 (17.7)	0.153
EVLP use	82 (35.2)	33 (30.3)	49 (39.5)	0.169
Total ischemic time, minutes	684 [561–1,009]	658 [543–941]	736 [573–1,049]	0.071
Post-transplant				
Extubation time, hours	50.16 [33.60–92.40]	48.48 [31.20–72.00]	57.00 [35.70, 106.26]	0.163
ICU length of stay, days	4.0 [3.0–9.0]	4.0 [3.0–9.0]	4.5 [2.0–9.0]	0.934
Transplant length of stay, days	22.95 [16.11–35.51]	21.85 [15.16–35.48]	23.15 [16.92, 35.67]	0.380

Binary and categorical variables are shown as number and (percentage) of the group total and compared using Fisher’s Exact test. Continuous variables are shown as median and [IQR] and compared using the Wilcoxon rank-sum test. BMI, Body mass index; DCD, donation after cardiac death; PRA, Panel of reactive antibodies; PLEX, plasmapheresis; EVLP, *Ex-Vivo* Lung Perfusion.

^*^Actual crossmatch after 2018 performed only for subjects with a positive current or historic virtual crossmatch. For crossmatches performed after this time, actual crossmatch was labeled as “negative” if virtual and historic crossmatch was negative.

Bold values are p valued below the significance threshold of 0.05.

### 3.3 Time to best achieved cPFT and oscillometry values

**Figure 2:**
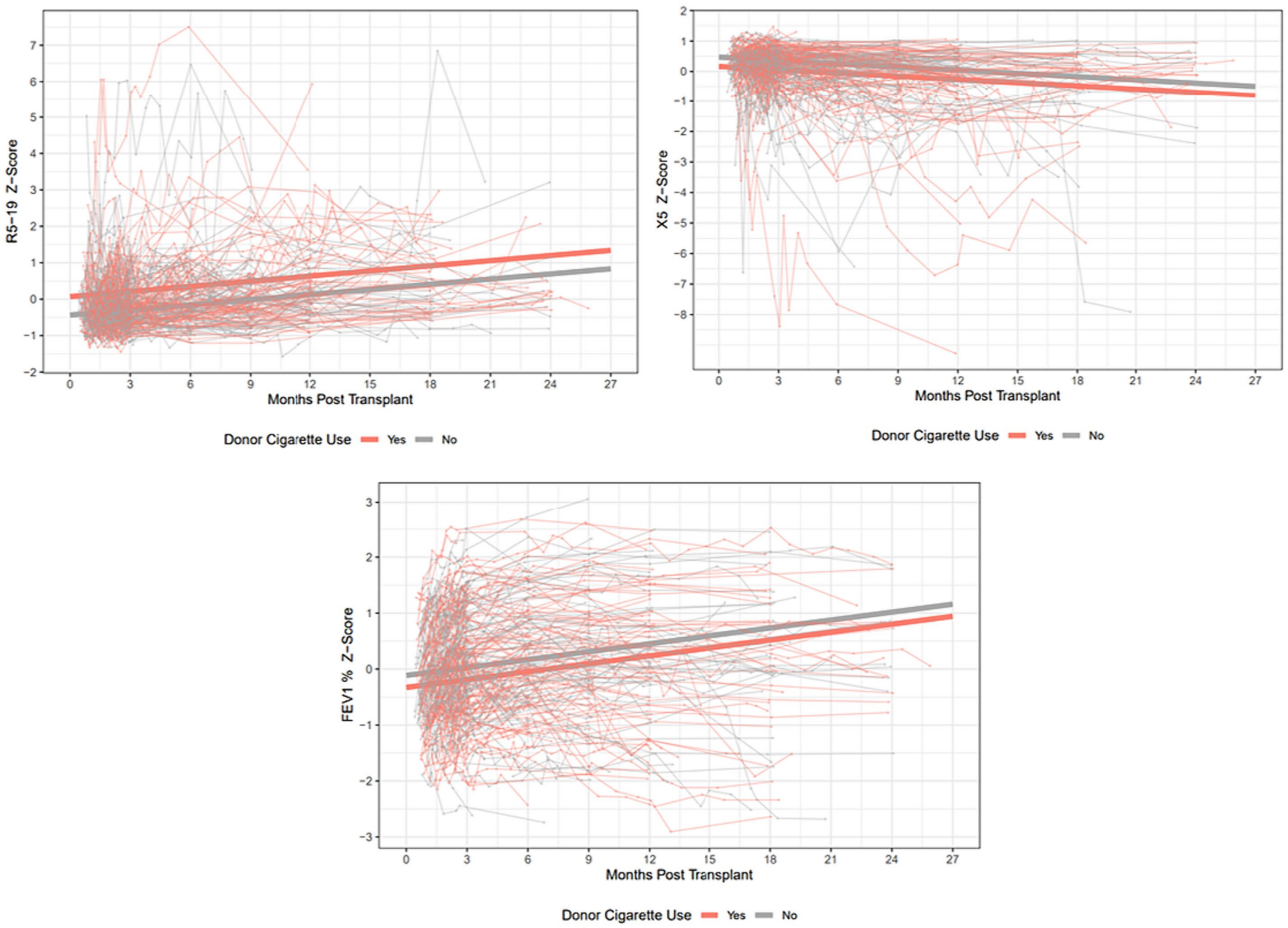
Spaghetti plots showing individual trajectories over time for R_5-19_, X_5_ and FEV_1_% predicted, with group trajectories estimated by mixed linear effects models. Z-scores are plotted to allow ease of comparison between the 3 parameters. R_5-19_, difference in resistance at 5 and 19 Hz; X_5_, reactance at 5 Hz; FEV_1_, forced expiratory volume in 1 s.

The median times to peak FEV_1_ and FVC in our cohort were 160 and 186 days, respectively. The median times to best achieved R_5_, R_5-19_, X_5_ and AX were 62, 67, 86 and 93 days, respectively. The best respiratory mechanics are the lowest values for R_5_, R_5-19_ and AX, and the highest, or least-negative value for X_5_. Spaghetti plots showing individual trajectories of R_5-19_, X_5_ and FEV_1_, and the estimated trajectories for the smoking and non-smoking group, are shown in Figure 2.

### 3.4 Association between donor smoking history and oscillometry

A total of 2,658 paired oscillometry-cPFT tests were included; 1,213 in non-smoking and 1,445 in smoking donor group. DLCO measurements are only performed at some PFT lab visits, therefore only 622 measurements were available for inclusion in the analysis.

In multivariable analysis, donor smoking compared to no smoking was found to be associated with all the oscillometry parameters examined except R_5_ and R_5_z. Donor smoking was associated with a 0.5 standard-deviation (SD) increase in R_5-19_ (95% CI 0.15–0.85, *p* = 0.006), a 0.30 SD decrease in X_5_ (95% CI–0.58 – –0.02, *p* = 0.035) and a 0.38 SD increase in AX (95%CI 0.05–0.71, *p* = 0.023). R_5_ was 0.34 standard deviations higher in the smoking group, but this association did not reach the pre-specified statistical significance level (*p* = 0.053, 95%CI 0.0–0.69). Donor smoking status did not have a significant association with any cPFT parameters (Table 3). Among the other covariates included in the models, time post-transplant was associated with all outcomes; donor age was associated with FEV_1_, FEV_1_/FVC and RV/TLC; underlying diagnosis was associated with all cPFT parameters and with X_5_; recipient height was associated with DLCO; R_5_, X_5_ and AX; and weight was associated with X5. Detailed results of all multivariable models are shown in Supplementary Table S1.

**Table 3:** Summary of multivariable donor smoking associations with cPFT and oscillometry outcomes.

	Donor smoking = yes		Donor smoking (pack years)	
Outcome	Adjusted regression coefficient (95% CI)	*p*	Adjusted regression coefficient (95% CI)	*p*
FEV_1_% predicted	−0.28 (−0.63, 0.07)	0.120	−0.07 (−0.20, 0.06)	0.321
FEV_1_/FVC %	−0.08 (−0.27, 0.11)	0.426	−0.10 (−0.23, 0.03)	0.142
TLC % predicted	0.12 (−0.09, 0.33)	0.254	−0.03 (−0.17, 0.11)	0.670
RV/TLC %	0.10 (−0.07, 0.27)	0.238	0.05 (−0.17, 0.27)	0.670
DLCO (mL/min/mmHg)^*^	−0.16 (−0.36, 0.04)	0.109	−0.01 (−0.15, 0.13)	0.890
R_5_ (cmH_2_O/L/s)	0.34 (0.00, 0.69)	0.053	0.15 (0.02, 0.28)	**0.021**
R_5_z	0.36 (−0.03, 0.76)	0.073	0.36 (0.03, 0.69)	**0.037**
R_5-19_ (cmH_2_O/L/s)	0.50 (0.15, 0.85)	**0.006**	0.19 (0.07, 0.32)	**0.004**
X_5_ (cmH_2_O/L/s)	−0.30 (−0.58,-0.02)	**0.035**	−0.13 (−0.24,-0.03)	**0.015**
X_5_z	−0.45 (−0.79,-0.11)	**0.010**	−0.39 (−0.70,-0.09)	**0.012**
AX (cmH_2_O/L)	0.38 (0.05, 0.71)	**0.023**	0.37 (0.10, 0.64)	**0.008**

Regression coefficients, representing the increase in number of standard deviations of the parameter being tested, 95% CI and associated *p*-values are shown comparing positive donor smoking history with negative, and for severity of smoking exposure in 10-pack-year increments. Associations have been adjusted for potential confounding variables, with models constructed separately for each outcome. Complete model results are shown in Supplementary Tables S1, S2. FEV_1_, forced expiratory volume in 1 s; FCV, forced vital capacity; TLC, total lung capacity; RV, residual volume; DLCO, diffusion capacity for carbon monoxide; R_5_, resistance at 5 Hz; R_5_z, z-score for R_5_; R_5-19_, difference between resistance at 5 Hz and 19 Hz; X_5_, reactance at 5 Hz; X_5_z, z-score for X_5_; AX, area under the reactance curve.

^*^DLCO measurements were only performed on some of the PFT lab visits, therefore, the total number of DLCO measurements included in the analysis was 662.

Bold values are p valued below the significance threshold of 0.05.

In addition to the above associations, sex match had a significant interaction with donor smoking status, affecting the association between donor smoking status and oscillometry outcomes. The association between smoking and oscillometry metrics was observed in the F➔F group, but was non-significant in the other sex match groups once adjusted for the interaction. The association between donor smoking and X_5_ and AX was actually reversed in the M➔F group (Table 4).

**Table 4:** Association with donor smoking stratified by sex-match group.

	FEV_1_% predicted	R_5_	R_5_z	R_5-19_	X_5_	X_5_z	AX
F➔F	−0.28 (−0.63, 0.07)	0.34 (0.00, 0.69)	0.36 (−0.03, 0.76)	0.50 (0.15, 0.85)**	−0.30 (−0.58,-0.02)*	−0.45 (−0.79,-0.11)**	0.38 (0.05, 0.71)*
M➔F	0.22 (−0.29, 0.72)	−0.45 (−0.95, 0.04)	−0.46 (−1.03, 0.10)	−0.41 (−0.92, 0.10)	0.46 (0.06, 0.86)*	0.42 (−0.07, 0.91)	−0.60 (−1.08,-0.13)*
F➔M	−0.91 (−1.56,-0.26)**	0.17 (−0.47, 0.81)	0.24 (−0.48, 0.96)	0.00 (−0.65, 0.65)	−0.02 (−0.52, 0.48)	−0.18 (−0.80, 0.44)	−0.08 (−0.68, 0.53)
M➔M	0.11 (−0.20, 0.42)	−0.07 (−0.37, 0.24)	−0.24 (−0.59, 0.10)	−0.13 (−0.44, 0.18)	0.14 (−0.10, 0.38)	0.29 (−0.01, 0.58)	−0.10 (−0.39, 0.18)

Regression coefficient, representing the increase by number of standard deviations of the parameter being tested conferred by a history of smoking in the donor, and 95% CI shown. Cells highlighted in bold indicate a deleterious effect of smoking in the specified sex match group. Cells highlighted in italics indicate an improvement in the parameter associated with donor smoking in the specified sex match group. Other adjustment variables for each outcome parameter are shown in Supplementary Table S1. Sex-match shown as donor ➔ recipient. FEV_1_, forced expiratory volume in 1 s; R_5_, resistance at 5 Hz; R_5_z, z-score for R_5_; R_5-19_, difference between resistance at 5 Hz and 19 Hz; X_5_, reactance at 5 Hz; X_5_z, z-score for X_5_; AX, area under the reactance curve.

*0.01 ≤ *p* < 0.05; **p < 0.01.

In the smoking donor group (*n* = 124), a significant association was seen between increased donor smoking exposure, quantified by pack years, and all oscillometry outcomes, with higher R_5_, R_5-19_, R_5_z and AX, and more negative X_5_ and X_5_z. No associations were observed between increasing donor smoking exposure and cPFT outcomes (Table 3).

The interaction with sex match was less prominent when donor smoking was considered as a continuous variable. The sex match interaction was observed for RV/TLC and X_5_z. The results for each sex-match group, after adjustment for the interaction, are shown in Table 5.

**Table 5:** Associations between donor smoking as a continuous variable, and outcomes stratified by sex-match group.

	RV/TLC	R_5_z	X_5_z	AX
F➔F	0.05 (−0.17, 0.27)	0.36 (0.03, 0.69)*	−0.39 (−0.70,-0.09)*	0.37 (0.10, 0.64)**
M➔F	0.42 (0.13, 0.72)**	0.13 (−0.28, 0.55)	−0.16 (−0.51, 0.19)	0.04 (−0.28, 0.37)
F➔M	−0.03 (−0.22, 0.15)	−0.01 (−0.27, 0.25)	0.04 (−0.20, 0.28)	0.05 (−0.17, 0.27)
M➔M	0.02 (−0.16, 0.19)	0.12 (−0.16, 0.41)	−0.17 (−0.42, 0.08)	0.15 (−0.08, 0.37)

Regression coefficient representing the increase in number of standard deviations of the parameter being tested, and 95% CI shown per 10-pack-year increase. Other adjustment variables for each outcome parameter are shown in Supplementary Table S2. Sex-match shown as donor ➔ recipient. R_5_, resistance at 5 Hz; R_5_z, z-score for R_5_; R_5-19_, difference between resistance at 5 Hz and 19 Hz; X_5_, reactance at 5 Hz; X_5_z, z-score for X_5_; AX, area under the reactance curve.

*0.01 ≤ *p* < 0.05; **p < 0.01.

Bold values are p valued below the significance threshold of 0.05.

### 3.5 CLAD

**Figure 3:**
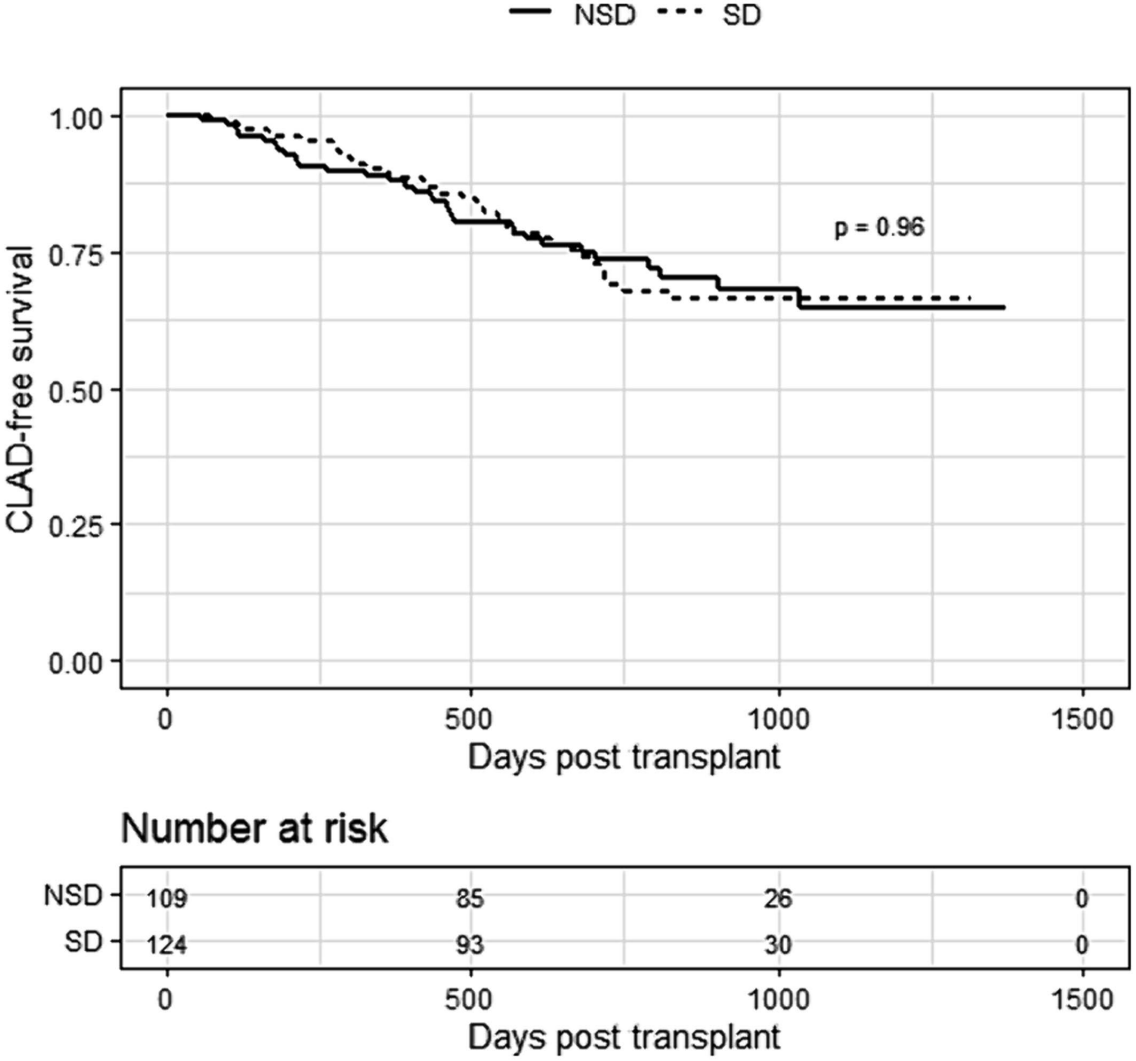
Kaplan–Meier curves for CLAD-free survival in lung transplant recipients from smoking donors (SD) and non-smoking donors (NSD). Log-rank *p*-value shown.

Over the study period, 15.6% of patients in the non-smoking group and 16.1% of patients in the smoking group developed CLAD. Kaplan-Myer curves comparing CLAD-free survival between the groups are shown in Figure 3. There was no significant difference between the survival curves (

*p* = 0.96).

## 4 Discussion

These results demonstrate a clear association between donor smoking history and abnormal oscillometry that reflects abnormal respiratory mechanics of the graft following lung transplantation, with a dose effect from increasing donor smoke exposure. The elevated R_5_, R_5_z, R_5-19_ and AX reflect increases in total respiratory resistance and ventilatory inhomogeneity, while lower X_5_ and X_5_z reflect loss of elastic recoil and increased lung stiffness (10, 34, 35). The association was not seen with conventional measures of post-transplant lung function such as FEV_1_, FEV_1_/FVC ratio and DLCO.

Oscillometry measures lung mechanics independently of patient effort and is sensitive to ventilatory heterogeneity in the lung periphery, reduced airway caliber and loss of tissue elastance (10, 34, 35). For these reasons, it may be a better indicator of early graft function, since much of the rapid increase in FEV_1_ and FVC, which is seen in the first few months post-transplant, may be attributable to improvement in physical conditioning rather than tissue quality. This hypothesis is supported by the observation that the baseline FEV_1_ value is often achieved 12 months or more post-transplant (5, 36), whereas oscillometry values stabilize earlier. In our cohort, the median time to best achieved FEV_1_ was more than double the time to best R_5_ and R_5-19_, and double the time to best X_5_ and AX. Our study also lends support to the utility of oscillometry for detecting subtle lung function abnormalities in the absence of clinical symptoms, which may be useful for early detection of conditions such as antibody-mediated rejection, which can have long-term consequences for graft function and development of CLAD. This is particularly important in the early post-transplant period when the expected overall upward trajectory of FEV_1_ can mask potentially significant graft dysfunction.

We did not find a significant difference in CLAD-free survival. However, the follow-up period is short, with a median of 356 days while the median CLAD onset time is 3–5 years post-transplant (37–40). We included patients with a very short duration of follow-up, starting as low as 3 months. As such, many of the included patients would not yet have reached their best post-transplant FEV_1_. A follow-up period of at least 12–18 months is required for to determine final baseline FEV_1_, though CLAD can still be diagnosed if an early drop in FEV_1_ occurs without subsequent recovery (5). The short duration of follow-up means that we were unable to determine the prevalence of BLAD in our cohort. Donor smoking has been identified as a risk factor for BLAD (4, 6), and BLAD patients exhibit a similar pattern of abnormalities to those seen in the smoking donor group in our study, with higher R_5-19_, R_5_, R_5_z and AX, and lower X_5_ and X_5_z (23). The role of oscillometry in predicting BLAD is an important subject that warrants further investigation.

The finding of an interaction between donor and recipient sex match and the effect of donor smoking history complicates the results, with an association between smoking and worse oscillometry outcomes seen in the reference (F➔F) group, but attenuated in the other groups, and even reversed for X_5_ and AX in the M➔F group. This interaction was less prominent when smoking was quantified by pack-years, but still affected R_5_z, X_5_z and AX in the F➔F group. The pack-year analysis was conducted in a smaller group of patients, and therefore may lack statistical power to observe the effects for a small sample size. Furthermore, the quantification of donor pack-years is imprecise, as this is estimated by the donor’s family during the donation process.

Female sex increases the deleterious effect of smoking with earlier onset of and worse outcomes from COPD (41, 42). Compared to men, women with COPD report more severe symptoms and poorer quality of life for similar age, FEV_1_, smoking history and proportion of emphysema on CT (43). This is in keeping with our finding that the deleterious effect of smoking is observed predominantly in female donors with female recipients. While the effect was not observed in the female donor/male recipient group after adjustment for the interaction effect, it must be noted that this was also the smallest of the 4 groups, encompassing just 28 participants.

Previous studies of sex matching and lung transplant outcomes have shown mixed results, and the topic remains controversial. Most studies focused on survival or CLAD. None looked specifically at the interaction between sex match and smoking. Minambres et al. (44) identified a non-significant trend toward increased mortality with female donors in a univariate analysis encompassing 152 transplants. Thabut et al. (45) also reported decreased long-term survival in the F➔M group, with an overall hazard ratio for mortality in female donors of 1.45. Roberts et al. (46) found a survival benefit for sex-mismatched pairs and improved survival for female recipients, while an analysis of ISHLT registry data (47) found that 90-day mortality was higher in the M➔F group and a lower in F➔F. Another ISHLT registry analysis found increase hazard of 10-year mortality F➔M and M➔F compared to F➔F (48). This more recent registry report focused on donor-recipient size mismatch, and identified an association between undersized organs and lower 1 and 5-year survival. As expected, undersizing was more frequent in the F➔M group, but the M➔F group also has worse long-term survival. Size mismatching offers an intuitively simple explanation for any discrepancies found, but the conflicting nature of existing data on sex matching and outcomes indicates this area requires further exploration, and that size matching is not the only factor influencing this relationship. The current study had not set out to explore size matching and this was not taken into account. However, the fact that the association between smoking and poorer oscillometry metrics was seen predominantly in the F➔F group, suggests that size matching is unlikely to be the only explanation for the observed results. A thorough assessment of size mismatch and its relationship with sex match needs to be explored in a future study with a larger cohort. Overall, the results of our study indicate a complex relationship between donor smoking history, sex match, and lung function post-transplant that warrants further investigation.

Our study has several limitations. Due to the challenge of quantifying smoking exposure in a deceased donor, the number of pack years is an approximate value. Second, the current oscillometry parameters do not have established predicted values across the full range of frequencies. Oostveen et al. (49) established impedance values across lower frequencies ≤14 Hz for reactance and all frequencies except 20 and 25 Hz for resistance, with good reproducibility between centers and devices, and provided prediction equations for R_5_ and X_5_. This allowed us to examine z-scores for these two parameters. The group comparisons on multivariable analysis were concordant, showing the same significant differences when analysing the raw values and the z-scores for R_5_ and X_5_.

Due to the current paucity of information about post-transplant lung mechanics when measured by oscillometry, variable selection for the multivariable analysis posed a significant challenge. We adopted a data-driven approach to variable selection, and some variables which would usually be included in models addressing post-transplant outcomes, such as underlying disease, were excluded from some models, as directed by the backwards stepwise elimination process. We also opted not to correct for multiple comparisons, given the exploratory nature of the study, the modest cohort size and the relatively small number of outcome metrics being tested. However, it is encouraging to note that our results demonstrated associations between donor smoking and the majority of the oscillometry metrics, and no association with cPFT metrics. Future analyses, utilizing larger groups of subjects and longer follow-up, will tease out specific parameters which may have the strongest associations with donor smoking or other potential insults to the allograft.

Our study included 22 subjects whose last cPFT/Oscillometry measurement occurred less than 3 months post-transplant. Although we excluded those patients who died within 3 months of transplant, or who had been transplanted less than 3 months before the census date of March 15, 2020, some patients stopped undergoing testing before the 3-month mark either due to complications preventing further routine testing, or due to temporary closure of the PFT laboratory due to the COVID-19 pandemic. We opted not to exclude these patients, because despite the short duration of follow-up, each of them still contributed several data points to the analysis. Furthermore, as the highest incidence of mild–moderate rejection episodes occurs within the first 3 months (50, 51), these readings provide valuable data on lung function fluctuations during this high-risk period.

### 4.1 Conclusion

Our study provides further evidence for the utility of oscillometry in detecting subtle lung function abnormalities which are undetectable by conventional pulmonary function testing. Oscillometry is more sensitive to small airways resistance and less influenced by musculoskeletal deconditioning, and can detect abnormalities in the early-post-transplant lung function of recipients of grafts from smoking donors. Furthermore, we have demonstrated a complex interaction between donor and recipient sex match and donor smoking history, a finding which warrants further study. A longer period of follow-up will also be necessary to determine whether these early differences in graft function are associated with long-term survival and CLAD.

## Data availability statement

The raw data supporting the conclusions of this article will be made available by the authors, without undue reservation.

## Ethics statement

The studies involving humans were approved by University Health Network Research Ethics Board. The studies were conducted in accordance with the local legislation and institutional requirements. The participants provided their written informed consent to participate in this study.

## Author contributions

NB: Conceptualization, Data curation, Formal analysis, Investigation, Methodology, Writing – original draft, Writing – review & editing. AC: Formal analysis, Investigation, Methodology, Writing – original draft, Writing – review & editing. JM: Investigation, Methodology, Supervision, Writing – review & editing. AV: Data curation, Writing – review & editing. JW: Data curation, Methodology, Supervision, Writing – review & editing. RG: Data curation, Resources, Software, Writing – review & editing.TM: Formal analysis, Investigation, Methodology, Writing – review & editing. CR: Data curation, Resources, Supervision, Writing – review & editing. C-WC: Conceptualization, Funding acquisition, Investigation, Methodology, Project administration, Supervision, Writing – review & editing.
