# Supplementary material for: Effects of donor smoking history on early post-transplant lung function measured by oscillometry
Source: Front Med (Lausanne). 2024 Apr 9;11:1328395. doi: 10.3389/fmed.2024.1328395 (PMC11037252; doi:10.3389/fmed.2024.1328395)
Supplement: Supplementary file 1 [file Data_Sheet_1.docx]

***Supplementary material***

**Effects of donor smoking history on early post-transplant lung function measured by oscillometry**

**Belousova, Natalia, Cheng, Albert, Matelski, John, Wu, Joyce K.Y., Ghany, Rasheed, Martinu, Tereza, Ryan, Clodagh M., Vasileva, Anastasiia, Chow, C.W**.

Contents:

| Supplementary Figure S1 | Page 2 |
| --- | --- |
| Supplementary Table S1 | Page 3 |
| Supplementary Table S2 | Page 4 |

**Supplementary Figure S1**: Heatmaps showing additive and interactive effects between baseline parameters and (A) donor smoking or (B) total donor smoking exposure in pack years for each outcome of interest.

Final multivariable models were constructed including only those parameters which had an additive or an interactive effect.

A.


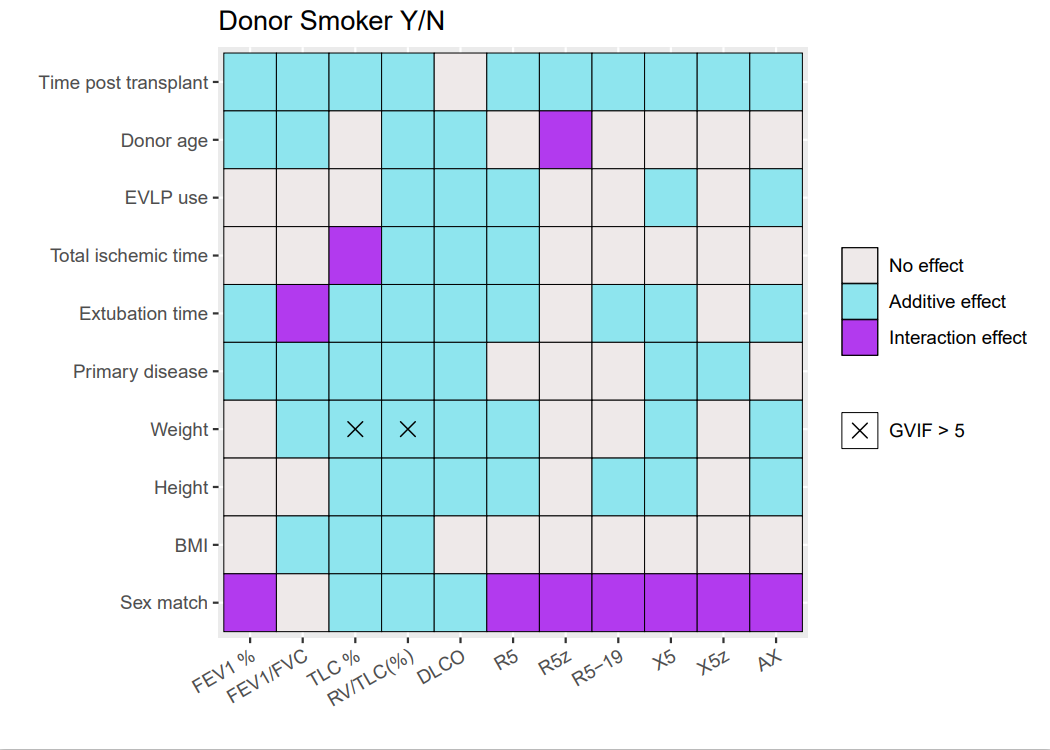


B.


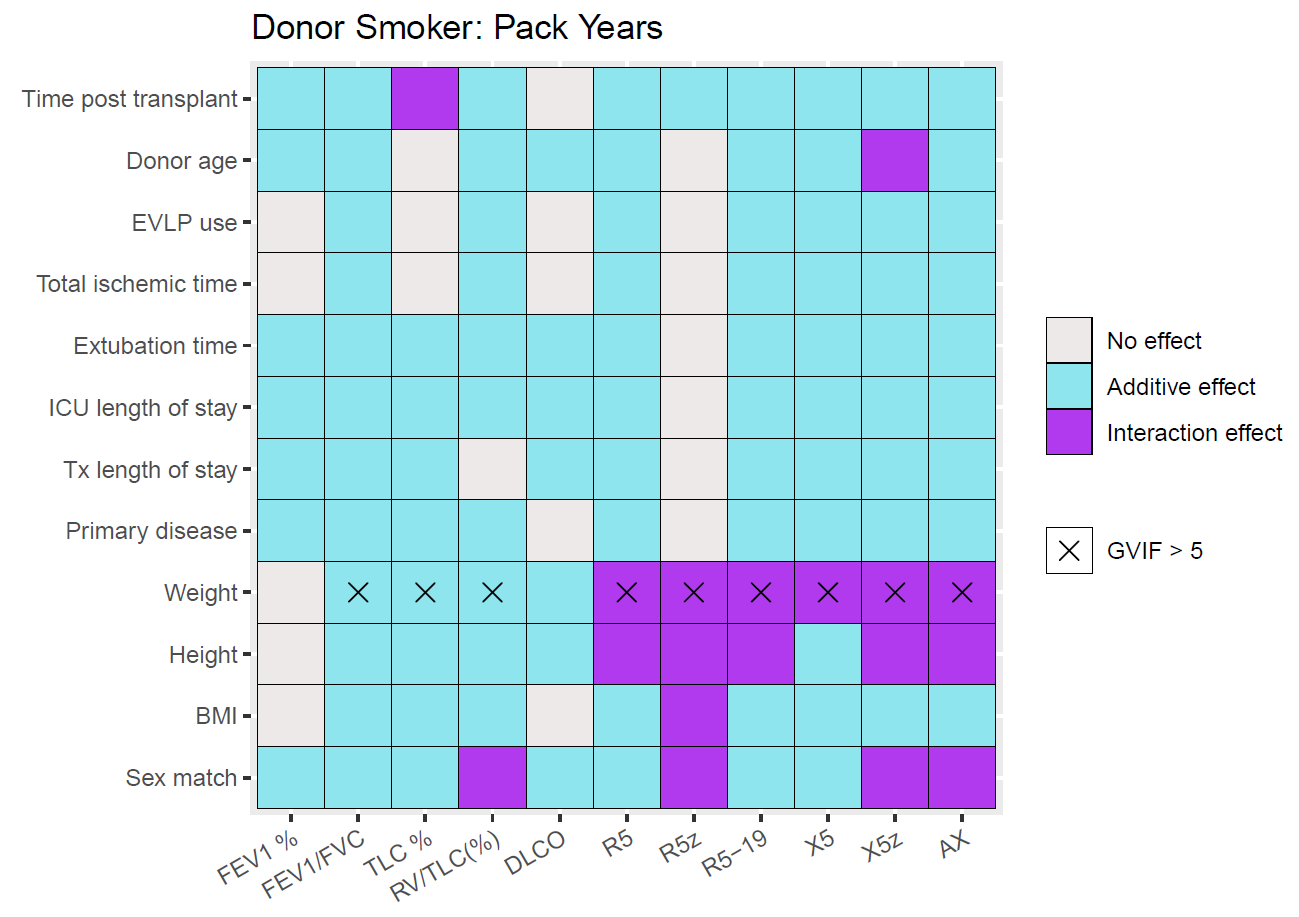


**Supplementary Table S1.** Complete multivaraible linear regression models for association between donor smoking history on PFT and oscillometry outcomes.

|  | FEV1 % predicted | FEV1/FVC | TLC % predicted | RV/TLC(%) | DLCO | R5 | R5z | R5-19 | X5 | X5z | AX |
| --- | --- | --- | --- | --- | --- | --- | --- | --- | --- | --- | --- |
| Donor smoking | -0.28 (-0.63, 0.07) | -0.08 (-0.27, 0.11) | 0.12 (-0.09, 0.33) | 0.10 (-0.07, 0.27) | -0.16 (-0.36, 0.04) | 0.34 ( 0.00, 0.69) | 0.36 (-0.03, 0.76) | 0.50 ( 0.15, 0.85)** | -0.30 (-0.58,-0.02)* | -0.45 (-0.79,-0.11)** | 0.38 ( 0.05, 0.71)* |
| Months post transplant | 0.05 ( 0.03, 0.06)** | -0.07 (-0.08,-0.06)** | 0.05 ( 0.04, 0.06)** | -0.06 (-0.08,-0.05)** | -0.26 (-0.36,-0.16)** | 0.05 ( 0.04, 0.06)** | 0.03 ( 0.02, 0.03)** | 0.05 ( 0.04, 0.06)** | -0.04 (-0.05,-0.02)** | -0.03 (-0.04,-0.01)** | 0.03 ( 0.02, 0.04)** |
| Donor age (yrs) | -0.16 (-0.26,-0.06)** | -0.27 (-0.37,-0.18)** |  | 0.13 ( 0.04, 0.21)** | 0.13 (-0.25, 0.52) |  | -0.14 (-0.28, 0.00) |  |  |  |  |
| EVLP use |  |  |  | -0.12 (-0.42, 0.18) | -0.07 (-0.26, 0.11) | -0.16 (-0.49, 0.17) |  |  | 0.08 (-0.09, 0.25) |  | -0.12 (-0.32, 0.07) |
| Total ischemic time (hrs) |  |  | -0.28 (-0.43,-0.14)** | 0.00 (-0.15, 0.14) | -0.14 (-0.24,-0.03)* | -0.02 (-0.18, 0.13) |  |  |  |  |  |
| Extubation time (hrs) | -0.19 (-0.30,-0.08)** | 0.08 (-0.07, 0.24) | -0.18 (-0.29,-0.06)** | 0.13 ( 0.04, 0.22)** | -0.16 (-0.36, 0.04) | 0.10 (-0.01, 0.20) |  | 0.12 ( 0.02, 0.23)* | -0.11 (-0.20,-0.03)* |  | 0.12 ( 0.02, 0.22)* |
| Primary disease |  |  |  |  |  |  |  |  |  |  |  |
| CF | Ref | Ref | Ref | Ref | Ref |  |  |  | Ref | Ref |  |
| Emphysema | 0.45 ( 0.12, 0.79)** | 0.41 ( 0.07, 0.74)* | 0.09 (-0.27, 0.44) | 0.91 ( 0.62, 1.21)** | -0.10 (-0.42, 0.23) |  |  |  | -0.17 (-0.43, 0.10) | -0.06 (-0.38, 0.26) |  |
| ILD | -0.14 (-0.48, 0.19) | -0.26 (-0.61, 0.09) | -0.64 (-1.02,-0.27)** | 0.45 ( 0.14, 0.76)** | -0.41 (-0.77,-0.06)* |  |  |  | -0.45 (-0.74,-0.17)** | -0.34 (-0.65,-0.03)* |  |
| Other | -0.34 (-0.72, 0.04) | -0.08 (-0.45, 0.29) | -0.24 (-0.64, 0.15) | 0.55 ( 0.22, 0.87)** | -0.27 (-0.64, 0.10) |  |  |  | -0.14 (-0.44, 0.16) | -0.12 (-0.48, 0.24) |  |
| Weight |  | -0.10 (-0.29, 0.09) |  |  | -0.03 (-0.17, 0.11) | 0.07 (-0.06, 0.20) |  |  | 0.13 ( 0.02, 0.24)* |  | 0.02 (-0.10, 0.14) |
| Height |  |  | -0.12 (-0.27, 0.04) | -0.10 (-0.22, 0.03) | 0.35 ( 0.18, 0.51)** | -0.26 (-0.42,-0.10)** |  | -0.07 (-0.22, 0.08) | 0.15 ( 0.01, 0.28)* |  | -0.16 (-0.32,-0.01)* |
| BMI |  | -0.11 (-0.30, 0.08) | -0.09 (-0.20, 0.03) | -0.30 (-0.39,-0.20)** |  |  |  |  |  |  |  |
| Sex match (D/R) |  |  |  |  |  |  |  |  |  |  |  |
| F/F | Ref | Ref | Ref | Ref | Ref | Ref | Ref | Ref | Ref | Ref | Ref |
| M/F | -0.24 (-0.66, 0.19) |  | 0.10 (-0.22, 0.42) | -0.06 (-0.33, 0.20) | 0.04 (-0.26, 0.34) | 0.51 ( 0.09, 0.93)* | 0.41 (-0.06, 0.89) | 0.63 ( 0.20, 1.06)** | -0.28 (-0.61, 0.06) | -0.39 (-0.80, 0.02) | 0.54 ( 0.15, 0.93)** |
| F/M | 0.51 (-0.10, 1.12) |  | -0.38 (-0.77, 0.00) | -0.28 (-0.59, 0.03) | 0.28 (-0.08, 0.63) | -0.36 (-0.98, 0.25) | -0.31 (-0.98, 0.36) | 0.12 (-0.50, 0.74) | 0.05 (-0.43, 0.53) | -0.01 (-0.58, 0.57) | 0.11 (-0.47, 0.68) |
| M/M | -0.12 (-0.45, 0.21) |  | -0.18 (-0.54, 0.17) | -0.54 (-0.83,-0.25)** | 0.55 ( 0.22, 0.88)** | 0.02 (-0.38, 0.42) | 0.24 (-0.13, 0.60) | 0.25 (-0.16, 0.66) | -0.11 (-0.43, 0.21) | -0.35 (-0.66,-0.03)* | 0.16 (-0.21, 0.54) |
| Interaction terms |  |  |  |  |  |  |  |  |  |  |  |
| DS/FF | Ref |  |  |  |  | Ref | Ref | Ref | Ref | Ref | Ref |
| DS/MF | 0.49 (-0.12, 1.11) |  |  |  |  | -0.79 (-1.40,-0.19)* | -0.83 (-1.52,-0.13)* | -0.91 (-1.53,-0.29)** | 0.76 ( 0.27, 1.24)** | 0.87 ( 0.28, 1.47)** | -0.98 (-1.56,-0.41)** |
| DS/FM | -0.63 (-1.37, 0.11) |  |  |  |  | -0.17 (-0.90, 0.55) | -0.12 (-0.94, 0.70) | -0.50 (-1.24, 0.24) | 0.28 (-0.29, 0.85) | 0.27 (-0.44, 0.97) | -0.46 (-1.14, 0.23) |
| DS/MM | 0.39 (-0.08, 0.85) |  |  |  |  | -0.41 (-0.87, 0.05) | -0.61 (-1.13,-0.08)* | -0.63 (-1.10,-0.16)** | 0.44 ( 0.07, 0.80)* | 0.73 ( 0.29, 1.18)** | -0.48 (-0.91,-0.05)* |
| DS/extubation time |  | -0.18 (-0.37, 0.02) |  |  |  |  |  |  |  |  |  |
| DS/ischemic time |  |  | 0.28 ( 0.08, 0.48)** |  |  |  |  |  |  |  |  |
| DS/Donor age |  |  |  |  |  |  | 0.22 ( 0.00, 0.45) |  |  |  |  |

Regression coefficient (and 95% CI) shows change in standard deviations of the outcome metric per standard deviation increase for continuous variables, with the exception of months post transplant (increase per month), or by comparison with reference category for categorical variables. Tx: transplant; EVLP: Ex-Vivo Lung Perfusion; CF: Cystic Fibrosis; ILD: interstitial lung disease; DS: Donor smoking; FEV_1_: forced expiratory volume in 1 second; FCV: forced vital capacity; TLC: total lung capacity; RV: residual volume; DLCO: diffusion capacity for carbon monoxide; R_5_: resistance at 5Hz; R_5_z: z-score for R_5_; R_5-19_: difference between resistance at 5Hz and 19Hz; X_5_: reactance at 5Hz; X_5_z: z-score for X_5_; AX: area under the reactance curve.

* 0.01 ≤ p < 0.05; ** p < 0.01

**Supplementary Table S2.** Complete multivariable linear regression models for donor smoking history quantified in pack years, and PFT and oscillometry outcomes. Regression coefficient and 95% CI shows change in standard deviations of the outcome metric per standard deviation increase for continuous variables, with the exception of donor pack years (increase in 10 pack-year increments) and months post transplant (increase per month); or by comparison with reference category for categorical variables. Tx: transplant; EVLP: Ex-Vivo Lung Perfusion; CF: Cystic Fibrosis; ILD: interstitial lung disease; DS: Donor smoking; DPY: Donor pack years; FEV_1_: forced expiratory volume in 1 second; FCV: forced vital capacity; TLC: total lung capacity; RV: residual volume; DLCO: diffusion capacity for carbon monoxide; R_5_: resistance at 5Hz; R_5_z: z-score for R_5_; R_5-19_: difference between resistance at 5Hz and 19Hz; X_5_: reactance at 5Hz; X_5_z: z-score for X_5_; AX: area under the reactance curve. * 0.01 ≤ p < 0.05; ** p < 0.01

|  | FEV1 % predicted | FEV1/FVC(%) | TLC % predicted | RV/TLC(%) | DLCO | R5 | R5z | R5-19 | X5 | X5z | AX |
| --- | --- | --- | --- | --- | --- | --- | --- | --- | --- | --- | --- |
| DPY | -0.07 (-0.20, 0.06) | -0.10 (-0.23, 0.03) | -0.03 (-0.17, 0.11) | 0.05 (-0.17, 0.27) | -0.01 (-0.15, 0.13) | 0.15 ( 0.02, 0.28)* | 0.36 ( 0.03, 0.69)* | 0.19 ( 0.07, 0.32)** | -0.13 (-0.24,-0.03)* | -0.39 (-0.70,-0.09)* | 0.37 ( 0.10, 0.64)** |
| Time post transplant (yrs) | 0.04 ( 0.02, 0.06)** | -0.07 (-0.09,-0.05)** | 0.05 ( 0.03, 0.06)** | -0.06 (-0.09,-0.04)** |  | 0.05 ( 0.04, 0.07)** | 0.03 ( 0.02, 0.04)** | 0.05 ( 0.04, 0.07)** | -0.04 (-0.06,-0.02)** | -0.03 (-0.05,-0.01)** | 0.04 ( 0.02, 0.05)** |
| Donor age (yrs) | -0.12 (-0.28, 0.05) | -0.32 (-0.48,-0.16)** |  | 0.12 (-0.01, 0.25) | -0.26 (-0.43,-0.09)** | -0.05 (-0.21, 0.10) |  | -0.01 (-0.18, 0.15) | 0.06 (-0.07, 0.19) | 0.03 (-0.15, 0.21) | -0.06 (-0.20, 0.09) |
| EVLP use |  | -0.33 (-0.82, 0.17) |  | -0.19 (-0.63, 0.25) |  | -0.19 (-0.67, 0.29) |  | -0.13 (-0.62, 0.36) | 0.24 (-0.16, 0.65) | 0.00 (-0.49, 0.49) | -0.09 (-0.53, 0.36) |
| Total ischemic time (hrs) |  | 0.09 (-0.15, 0.33) |  | 0.02 (-0.20, 0.24) |  | -0.04 (-0.27, 0.20) |  | 0.04 (-0.20, 0.28) | -0.08 (-0.27, 0.12) | -0.01 (-0.24, 0.22) | -0.02 (-0.23, 0.19) |
| Extubation time (hrs) | -0.23 (-0.37,-0.08)** | -0.13 (-0.28, 0.02) | -0.16 (-0.32,-0.01)* | 0.12 ( 0.00, 0.24) | -0.16 (-0.32, 0.00)* | 0.16 ( 0.02, 0.31)* |  | 0.14 ( 0.00, 0.29) | -0.15 (-0.27,-0.03)* | -0.08 (-0.22, 0.07) | 0.13 ( 0.00, 0.27) |
| Primary disease |  |  |  |  |  |  |  |  |  |  |  |
| CF | Ref | Ref | Ref |  |  | Ref |  | Ref | Ref | Ref | Ref |
| Emphysema | 0.60 ( 0.14, 1.07)* | 0.32 (-0.18, 0.81) | 0.21 (-0.29, 0.71) | 0.71 ( 0.32, 1.09)** |  | 0.08 (-0.40, 0.56) |  | 0.51 ( 0.03, 1.00)* | -0.21 (-0.61, 0.18) | -0.38 (-0.85, 0.09) | 0.26 (-0.18, 0.69) |
| ILD | -0.04 (-0.50, 0.42) | -0.29 (-0.80, 0.23) | -0.61 (-1.13,-0.09)* | 0.24 (-0.17, 0.65) |  | -0.03 (-0.53, 0.47) |  | 0.44 (-0.07, 0.94) | -0.49 (-0.90,-0.07)* | -0.76 (-1.25,-0.28)** | 0.44 (-0.01, 0.90) |
| Other | -0.29 (-0.84, 0.26) | -0.09 (-0.64, 0.47) | -0.10 (-0.68, 0.48) | 0.35 (-0.09, 0.79) |  | -0.11 (-0.64, 0.43) |  | 0.11 (-0.44, 0.66) | -0.14 (-0.59, 0.31) | -0.18 (-0.70, 0.34) | 0.09 (-0.40, 0.57) |
| Weight |  |  |  |  | -0.06 (-0.25, 0.12) |  |  |  |  |  |  |
| Height |  | 0.00 (-0.20, 0.21) | -0.13 (-0.34, 0.08) | -0.16 (-0.32,-0.01)* | 0.32 ( 0.08, 0.56)* | -0.15 (-0.35, 0.05) | 0.11 (-0.11, 0.33) | -0.13 (-0.34, 0.07) | 0.15 (-0.01, 0.31) | -0.03 (-0.22, 0.16) | -0.10 (-0.27, 0.08) |
| BMI |  | -0.12 (-0.28, 0.03) | -0.01 (-0.17, 0.15) | -0.25 (-0.37,-0.12)** |  | -0.01 (-0.17, 0.14) | -0.14 (-0.30, 0.01) | 0.01 (-0.14, 0.17) | 0.16 ( 0.03, 0.28)* | 0.22 ( 0.07, 0.37)** | -0.09 (-0.22, 0.05) |
| Sex match (D/R) |  |  |  |  |  |  |  |  |  |  |  |
| F/F | Ref | Ref | Ref | Ref | Ref | Ref | Ref | Ref | Ref | Ref | Ref |
| M/F | 0.22 (-0.23, 0.67) | -0.02 (-0.48, 0.45) | 0.37 (-0.11, 0.85) | -0.14 (-0.52, 0.24) | 0.14 (-0.36, 0.65) | -0.42 (-0.87, 0.02) | -0.53 (-1.05,-0.02)* | -0.29 (-0.75, 0.17) | 0.57 ( 0.20, 0.95)** | 0.57 ( 0.12, 1.02)* | -0.57 (-0.98,-0.17)** |
| F/M | -0.15 (-0.58, 0.28) | -0.17 (-0.64, 0.31) | -0.42 (-0.91, 0.08) | -0.15 (-0.53, 0.23) | 0.22 (-0.27, 0.71) | -0.57 (-1.04,-0.11)* | -0.40 (-0.93, 0.13) | -0.38 (-0.85, 0.09) | 0.41 ( 0.02, 0.79)* | 0.23 (-0.22, 0.69) | -0.42 (-0.84, 0.00) |
| M/M | 0.16 (-0.19, 0.52) | -0.24 (-0.73, 0.25) | -0.19 (-0.68, 0.31) | -0.51 (-0.89,-0.13)** | 0.63 ( 0.13, 1.12)* | -0.45 (-0.92, 0.02) | -0.51 (-1.04, 0.01) | -0.33 (-0.80, 0.15) | 0.37 (-0.02, 0.76) | 0.33 (-0.13, 0.79) | -0.40 (-0.82, 0.02) |
| Interaction terms |  |  |  |  |  |  |  |  |  |  |  |
| DPY/FF |  |  |  | Ref |  |  | Ref |  |  | Ref | Ref |
| DPY/FM |  |  |  | 0.38 ( 0.01, 0.74)* |  |  | -0.23 (-0.73, 0.28) |  |  | 0.23 (-0.20, 0.67) | -0.33 (-0.72, 0.07) |
| DPY/MF |  |  |  | -0.08 (-0.38, 0.22) |  |  | -0.37 (-0.78, 0.05) |  |  | 0.44 ( 0.07, 0.80)* | -0.32 (-0.66, 0.02) |
| DPY/MM |  |  |  | -0.03 (-0.31, 0.24) |  |  | -0.23 (-0.72, 0.25) |  |  | 0.22 (-0.22, 0.66) | -0.22 (-0.61, 0.16) |
| DPY/years post tx |  |  | 0.01 ( 0.00, 0.02) |  |  |  |  |  |  |  |  |
| DPY/BMI |  |  |  |  |  |  | -0.10 (-0.27, 0.06) |  |  |  |  |
| DPY/height |  |  |  |  |  | -0.18 (-0.31,-0.05)** | -0.16 (-0.36, 0.03) | -0.23 (-0.37,-0.10)** |  | 0.09 (-0.08, 0.26) | -0.09 (-0.25, 0.07) |
| DPY/donor age |  |  |  |  |  |  |  |  |  | -0.08 (-0.28, 0.12) |  |
